# Buerger's Disease in the Testicle: A Case of Testicular Thromboangiitis Obliterans

**DOI:** 10.7759/cureus.37693

**Published:** 2023-04-17

**Authors:** Emma A Harwood, Andrew J Blazek, Stanley J Radio, Christopher M Deibert

**Affiliations:** 1 Division of Urologic Surgery, University of Nebraska Medical Center, Omaha, USA; 2 Department of Pathology and Microbiology, University of Nebraska Medical Center, Omaha, USA

**Keywords:** vasculitis, testicular lesion, testicular mass, thromboangiitis obliterans, buerger’s disease

## Abstract

Thromboangiitis obliterans (TAO), otherwise known as Buerger's disease, is a rare, non-atherosclerotic inflammatory vasculopathy that typically affects small and medium-sized arteries of the distal extremities. Smoking is believed to be integral to the pathogenesis, as TAO primarily affects young male smokers. The disease is characterized by extremity pain secondary to ischemia that may progress to ulceration, gangrene, and amputation. Involvement of the reproductive system is uncommon. Here, we offer a case of TAO presenting as a testicular mass lesion.

## Introduction

Thromboangiitis obliterans (TAO), otherwise known as Buerger\s disease, is a segmental, thrombotic, occlusive vasculitis of small and medium-sized arteries [1,2]. The exact cause of the disorder is not known. Atherosclerosis is not involved in the pathogenesis, but smoking is strongly related to the development and progression of the disease [3,4]. Tobacco smoke is the most common risk factor, but TAO may also develop in association with smoking marijuana or chewing tobacco [2,5]. This disorder most commonly develops in males under the age of 40 years, but it also occurs in females, teenagers, and the elderly [2,5,6]. The prevalence of Buerger\s is higher in those of Eastern European or Asian descent [7].

The disease is characterized by inflammation primarily of the tunica intima, sometimes spreading to the tunica media. An inflammatory thrombus composed of a variety of cells forms, ultimately occluding the involved vessel [7]. The inflammation may extend to adjacent veins and nerves. The most commonly affected vessels are those of the distal extremities, manifesting as rest pain in the extremities, digital ischemia, ulcerations, and gangrene leading to amputation [4,8]. Claudication may occur with progression to more proximal vessels [5]. Raynaud\s phenomenon and superficial thrombophlebitis are other common findings [3,4]. Of note, cerebral, visceral, ocular, and coronary vessels may be affected, albeit rarely [7,9-12]. There have also been rare cases of Buerger\s involving the male reproductive organs, including the spermatic cord and the testis [13-18]. In this report, we present a rare case of a testicular lesion that proved to be thromboangiitis obliterans in the testicle.

## Case presentation

A 40-year-old man presented to the emergency department with two weeks of progressive pain in his left testicle. He denied any overlying skin changes, fevers, chills, or urinary symptoms. He denied prior pain or swelling in the testicle and denied a history of trauma to the area. He had no significant past medical or surgical history and was not taking any chronic medications. The patient has never been a biological father. He reported active marijuana use and a 25-pack-year history of smoking tobacco cigarettes.

On exam, the patient was found to have a normal penis with an orthotopic meatus. He had bilateral descended testicles without scrotal edema or erythema. The left testicle was non-tender but abnormally and universally hard without any discrete nodules or masses. The right testicle was normal without masses, lesions, or abnormalities.

**Figure 1:**
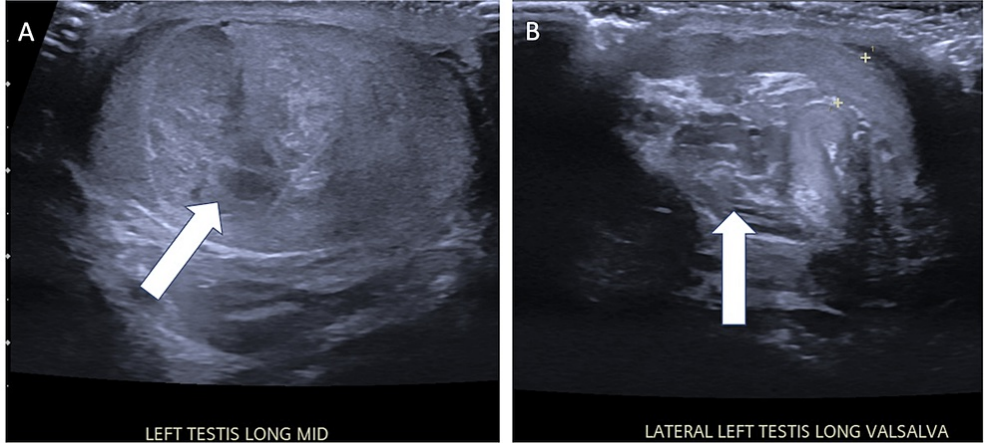
Left testis ultrasound Ultrasound of the left testis demonstrating a mass and associated tortuous vessels (arrows), which are commonly seen in thromboangiitis obliterans (Buerger's disease).

Ultrasound exam revealed the left testicle to be a large, solid, and heterogenous mass with increased blood flow concerning testicular malignancy versus heterogeneous hematoma (Figure 1). Given the concern for testicular cancer, contrast-enhanced computed tomography (CT) imaging was obtained in accordance with The National Comprehensive Cancer Network® (NCCN®) Clinical Practice Guidelines in Oncology (NCCN Guidelines®)[19]. CT confirmed a hypervascular testicular mass with no pelvic or inguinal lymphadenopathy. Tumor markers were within normal limits: alpha-fetoprotein was <1.3 ng/mL, lactate dehydrogenase was 252 u/l, and human chorionic gonadotrophin was <1 mIU/mL.

Given the inability to rule out malignancy, the patient subsequently underwent a left radical inguinal orchiectomy. A testicular prosthetic was then placed per patient preference.

**Figure 2:**
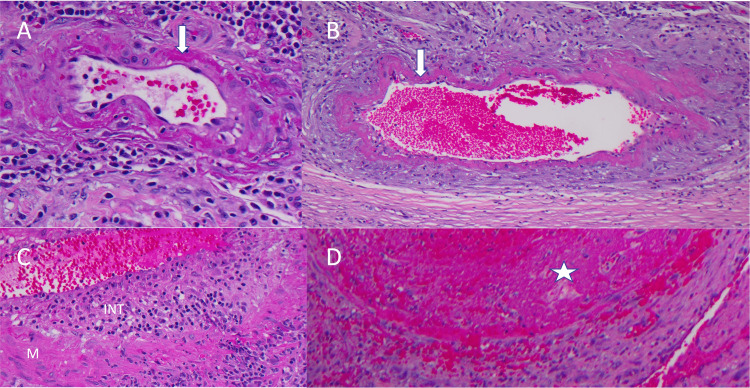
Histologic findings consistent with thromboangiitis obliterans (Buerger's disease) A: Fibrinoid necrosis (arrow) present in the intima and media of a small to medium-sized artery. B: Fibrinoid necrosis (arrow) present in medium-sized vein. C: Mixed inflammatory infiltrate involving intima (INT), media (M), and adventitia. D: Vein with luminal (star symbol) thrombus.

Pathologic tissue analysis was consistent with extensive organizing hemorrhage and negative for malignancy. Vascular changes included fibrinoid necrosis in the medial vessel layers and transmural inflammatory infiltrate composed of lymphocytes, macrophages (confirmed by CD68), and scattered neutrophils. Focal intravascular thrombi were seen. These histologic findings are consistent with an active vasculitis involving many arteries and a few veins confirmed by a dedicated cardiovascular pathologist (SJR). Given the patient's history of cannabis and tobacco use, the patient was ultimately diagnosed with thromboangiitis obliterans (Buerger's disease; Figure 2). We made a referral to rheumatology for further testing, but the patient was not interested. 

## Discussion

There are a small number of case reports describing TAO in the male reproductive system, including the spermatic cord, penis, and one prior report of TAO in the testicle [13-18]. Thus, our presented case is a rare entity, given the lack of distal extremity involvement and characteristic sequelae. Instead, the patient presented with a painful and abnormally firm left testicle. Given the concern for testicular malignancy on exam and imaging, a normal contralateral testicle, and recommendations for testicular masses in the NCCN Guidelines®[19], a biopsy was not done, and the patient subsequently underwent left radical inguinal orchiectomy.

The involvement of the male reproductive system raises the question of whether thromboangiitis obliterans affects fertility. One recent publication [20] demonstrated that impeded genital blood flow and a higher prevalence of antisperm antibodies in TAO patients may play a role in a patient's secondary infertility. The patient presented here had no children and did not undergo a semen analysis.

Thromboangiitis obliterans is a rare disease that can be challenging to diagnose. Depending on the presentation, the differential diagnosis may include autoimmune disorders such as systemic lupus erythematosus, antiphospholipid antibody syndrome, mixed connective tissue disease, or other systemic vasculitides [4,8]. The diagnosis is largely clinical and not commonly confirmed by biopsy because of the difficulty in accessing the affected vessels [2,4]. This case underscores the importance of a complete history and consideration of the whole clinical picture. Establishing a definitive diagnosis is crucial to proper counseling of patients, given that without cannabis and tobacco cessation, the disease will likely progress with increasing morbidity [2,3,5]. Symptoms may be managed medically with calcium channel blockers, prostaglandin analogs, or opioid pain relievers, but the vascular damage is irreversible [4].

## Conclusions

Buerger's disease involving the male reproductive system is a rare entity. The differential diagnosis of testicular malignancy should be included in any patient that presents with a testicular lesion, but history and clinical judgment are paramount to definitively diagnosing Buerger's disease. Smoking cessation remains the primary treatment. There are few cases of Buerger's disease involving the male reproductive system available in the literature, so additional reports will lead to a better understanding and management of similar cases.
